# Patient-reported outcomes in hip resurfacing versus conventional total hip arthroplasty: a register-based matched cohort study of 726 patients

**DOI:** 10.1080/17453674.2019.1604343

**Published:** 2019-04-18

**Authors:** Alexander Oxblom, Håkan Hedlund, Szilard Nemes, Harald Brismar, Li Felländer-Tsai, Ola Rolfson

**Affiliations:** a Divison of Orthopaedics and Biotechnology, CLINTEC, Karolinska Institutet;;; b Visby Lasarett;;; c Swedish Hip Arthroplasty Register, Gothenburg;;; d Department of Orthopaedics, Institute of Clinical Sciences, Sahlgrenska Academy University of Gothenburg, Sweden

## Abstract

Background and purpose — The theoretical mechanical advantages of metal-on-metal hip resurfacing (MoM-HR) compared with conventional total hip arthroplasty (THA) have been questioned. Studies including measures of patient-reported function, physical activity, or health-related quality of life have been sparse. We compared patient-reported outcomes in MoM-HR patients with a matched group of patients with conventional THA at 7 years post-surgery.

Patients and methods — Patients and patient data were retrieved from the Swedish Hip Arthroplasty Register. The case group, consisting of 363 patients with MoM-HR, was matched 1:1 with a control group, consisting of patients with a conventional THA. Patients were sent a postal patient-reported outcome measures (PROM) questionnaire including the Hip Disability and Osteoarthritis Outcome Score (HOOS), EQ-5D, and VAS pain. We used multivariable linear regression analyses to investigate the influence of prosthesis type.

Results — 569 patients (78%) returned the questionnaire with complete responses (299 MoM-HRs and 270 conventional THAs). MoM-HR was associated with better scores in HOOS function of daily living (4 percentage units) and HOOS function in sport and recreation (8 percentage units) subscales. Type of prosthesis did not influence HOOS quality of life, HOOS pain, HOOS symptoms, EQ-5D index, hip pain, or satisfaction as measured with visual analog scales.

Interpretation — At mean 7 years post-surgery, patients with hip resurfacing had somewhat better self-reported hip function than patients with conventional THA. The largest difference between groups was seen in the presumed most demanding subscale, i.e., function in sport and recreation.

Hip arthroplasty in young and active patients is an orthopedic challenge. In 2011, the Finnish Arthroplasty Register (Mäkelä et al. 2011) reported a 15-year prosthesis survival rate of about 70% in patients younger than 55 years operated with conventional total hip arthroplasty (THA) compared with about 90% in patients older than 60 years in the combined Nordic Arthroplasty Registers (Havelin et al. 2009). Young patients have higher expectations following THA (Scott et al. 2012) and are more active, a patient-factor highly related to polyethylene wear (Schmalzried et al. 2000). They are also more prone to participate in high-impact sports following THA (Williams et al. 2012), which has been correlated with both increased wear (Ollivier et al. 2012) and higher revision rates (Flugsrud et al. 2007). Alternative surface bearings and prosthesis designs have therefore been developed to meet the demands of younger patients.

Metal-on-metal hip resurfacing (MoM-HR) gained popularity in the mid-1990s due to advances in metallurgy and tribology, allowing manufacturing of thin acetabular cups accepting large-diameter components (Grigoris et al. 2006). It was believed that the wear-associated disadvantages seen with metal-on-polyethylene thereby could be solved. The method was expected to provide a sustainable arthroplasty for young and active patients with hip osteoarthritis (Amstutz and Le Duff 2012). Besides a bone-preserving surgical technique, MoM-HR was also claimed to restore hip mechanics with a better range of motion (Vail et al. 2006). However, there was a major setback when some MoM-HR implants and THAs with MoM articulations were reported to have unacceptably high failure rates (De Steiger et al. 2011, Smith et al. 2012). As a result, there was a dramatic decline in numbers of MoM-HR implanted worldwide and, in many countries, surgeons promptly stopped using the technique, due to perceived risks and the uncertainty regarding the long-term results of the implants (Cohen 2011).

There are, though, some long-term follow-ups of certain brands of MoM-HR implants with acceptable implant survival in a selected group of patients (Matharu et al. 2013). It is evident that cautious patient selection is crucial, quite apart from implant design and surgical technique (Daniel et al. 2014).

Reports on benefits of MoM-HR in terms of patient-reported function, physical activity, and health-related quality of life are sparse (Jiang et al. 2011). We compared patient-reported outcomes in patients operated with MoM-HR with a matched group of patients operated with conventional THA at mean 7 years post-surgery.

## Patients and methods

### Patient selection

This is an arthroplasty register-based matched cohort study. Patient data were retrieved from the Swedish Hip Arthroplasty Register. The case group, consisting of a consecutive group of all patients operated on with MoM-HR (all Birmingham Hip Resurfacing System, Smith & Nephew, Andover, Massachusetts, USA) at a single institution (Karolinska Huddinge) between the years 2002 and 2013, was matched 1:1 with a control group, consisting of patients with a conventional THA selected from the Register. In the case of bilateral MoM-HR (n = 105) or bilateral THA (n = 102) during the study period, we included data regarding the first operation. Patients deceased by December 2015 (n = 6) were excluded. The groups were matched by baseline characteristics: age, sex, surgical approach, year of surgery, and preoperative EQ-5D score when available.

### Outcome measures

726 patients (363 MoM-HRs, 363 conventional THAs) were selected for the study (Table 1). In December 2015, patients were invited to participate by mail and asked to complete a patient-reported outcome measures (PROM) questionnaire including the Hip Disability and Osteoarthritis Outcome Score (HOOS) (Nilsdotter et al. 2003), the EQ-5D (EuroQol Group 1990), hip pain measured with a visual analogue scale (VAS), and a VAS addressing satisfaction with the outcome of surgery.

**Table 1.** Patient demographics

Characteristics	Case group	Control group	p-value
Number of patients	363 (	363 (	
Women, n (%)	90 (25)	86 (24)	0.8
Age at primary			
operation, mean (SD)	52 (8.8)	51 (8.7)	0.7
Year of surgery, mean (SD)	2008 (2.9)	2008 (2.9)	0.9
Follow-up time, mean (SD)	7.3 (2.9)	7.3 (3.0)	0.9
Distribution of diagnoses, n (%)			0.7
Primary osteoarthritis	315 (87)	325 (90)	
Childhood hip disease	41 (11)	31 (8.5)	
Other hip joint disorders	7 (2.0)	7 (2.0)	
PROMs preoperatively, n	206 (	363 (	
VAS hip pain, mean (SD)	74 (16.4)	69 (18.4)	0.002
EQ-5D index, mean (SD)	0.52 (0.29)	0.43 (0.32)	0.001
Patients reoperated, n (%)	13 (3.6)	16 (4.4)	0.6

SD = standard deviation; PROMs = patient-reported outcome measures; VAS = visual analog scale; EQ-5D = EuroQol 5 dimensions.

In addition to the postal questionnaire we used information from the Swedish Hip Arthroplasty Register covering surgical data, demography, data on subsequent reoperations and, when available, pre- and postoperative PROMs data including hip pain and the EQ-5D (Garellick et al. 2015).

### Statistics

Subject-matter knowledge was used to identify and measure adjustment variables. The goal was to identify a sufficient set for confounding adjustment for prosthesis type. This set was defined as a set of non-descendant variables for prosthesis type that block all backdoor paths. Confounder identification was based on Rubin’s 3 conditions (Robins 1999, Greenland et al. 1999). By matching we constructed a subset of the population in which the background has the same distribution in both the MoM-HR and the conventional THA groups. In observational studies, there is no guarantee that the treatment groups are conditionally exchangeable given the exposure only. Matching generally exploits the conditional exchangeability; however, matching cases and controls does not achieve unconditional exchangeability. Ignoring the matching variables in a cohort study can leave bias if there are additional confounders, even with adjustment for the additional confounders (Sjölander and Greenland 2013). Based on these 2 facts the final analysis included the variables used for matching.

We identified age, sex, preoperative EQ-5D index, and time from surgery. Neither variable is on the path between the exposure and outcome and can block important backdoor paths (Figure 1, see Supplementary data). Using the Directed Acyclic Graph from Figure 1 and d-separation to infer associational statements (Textor et al. 2011) we could conclude that the minimal sufficient adjustment sets for estimating the direct effect and total effect is age, sex, and preoperative EQ-5D index. Time for surgery was included to reduce bias (Sjölander and Greenland 2013).

We used multivariable linear regression analyses to investigate the influence of prosthesis type (MoM-HR versus conventional THA) adjusting for age, sex, preoperative EQ-5D index, and time from surgery. R (R Core Team 2017) and IBM SPSS Statistics version 25 (IBM Corp, Armonk, NY, USA) were used for statistical analyses. Missing covariate data were imputed using full-conditional specification (FCS) multiple imputation with the inclusion of the outcomes and matching variables (Seaman and Keogh 2015). The imputed data were used as input for regression analyses and estimates from each imputed dataset were combined into 1 overall estimate and associated variance, incorporating both the within and between imputation variability using Rubin’s rules (Marshall et al. 2009). Regression estimates (coefficients) were reported with 95% confidence intervals (CI).

Observational studies are by nature subjected to unmeasured confounding. We postulate that the possible unblocked backdoor paths are weak. Confounding bias requires a strong confounder treatment and a strong confounder outcome association. Generally, baseline variables explain a low amount of variance of postoperative PROMs (Bengtsson et al. 2017, Nemes et al. 2018) and expectedly the residual confounding bias is low.

### Ethics, funding, and potential conflicts of interest

The study was approved by the Regional Ethical Review Board in Gothenburg (Dnr 407-14). This research did not receive any specific grants from commercial funding agencies or bodies. The study was supported by public funding from the Swedish Hip Arthroplasty Register and research funds from Stockholm County Council. No competing interest declared.

## Results

569 patients (78%) returned the questionnaire with complete responses. Mean follow-up time (F-U) was 7 years (IQR 2.2–13 years). The proportion of patients who had undergone any reoperation was similar between groups (Table 1). The preoperative demographics of the patients who did not answer the questionnaire did not demonstrate statistically significant difference from those who answered (Table 2, see Supplementary data).

**Table 2.** Non-respondent analysis

Characteristics	Respondents	Non-respondents	p-value
Number of ptients	569 (	157 (	
Women, n (%)	132 (23)	44 (28)	0.3
Cases, n (%)	299 (53)	64 (41)	0.01
Age at primary			
operation, mean (SD)	52 (8.6)	49 (9.0)	0.001
Year of surgery, mean (SD)	2008 (2.9)	2008 (2.8)	0.8
Distribution of diagnoses, n (%)			0.1
Primary osteoarthritis	508 (89)	132 (84)	
Childhood hip disease	49 (8.6)	23 (15)	
Other hip joint disorders	12 (2.1)	2 (1.3)	
PROMs preoperative			
VAS hip pain, mean (SD)	70 (18)	72 (19)	0.3
EQ-5D index, mean (SD)	0.49 (0.31)	0.40 (0.32)	0.006

SD = standard deviation; VAS = visual analog scale; EQ-5D = EuroQol 5 dimensions.

The case group had better unadjusted outcomes in all subscales of HOOS whereas EQ-5D index, VAS pain, and VAS satisfaction were equal between the groups (Table 3).

**Table 3.** Postoperative functional outcomes. Values are mean (SD)

Variables	Case group	Control group	p-value
HOOS index (%)			
Symptoms	85 (17)	83 (19)	0.09
Pain	90 (15)	87 (18)	0.01
ADL	90 (15)	84 (19)	< 0.001
Sport/Rec	77 (24)	68 (29)	< 0.001
QoL	77 (21)	74 (22)	0.07
EQ-5D index	0.90 (0.17)	0.87 (0.21)	0.2
VAS hip pain	11 (16)	12 (18)	0.4
VAS satisfaction	13 (20)	12 (21)	0.7

SD = standard deviation; HOOS = Hip Disability and Osteoarthritis Outcome Score; ADL = Activity in Daily Living; Sport/Rec = Sport and Recreation; QoL = Quality of Life; EQ-5D = EuroQol 5 dimensions; VAS = Visual analog scale.

**Figure 2.**
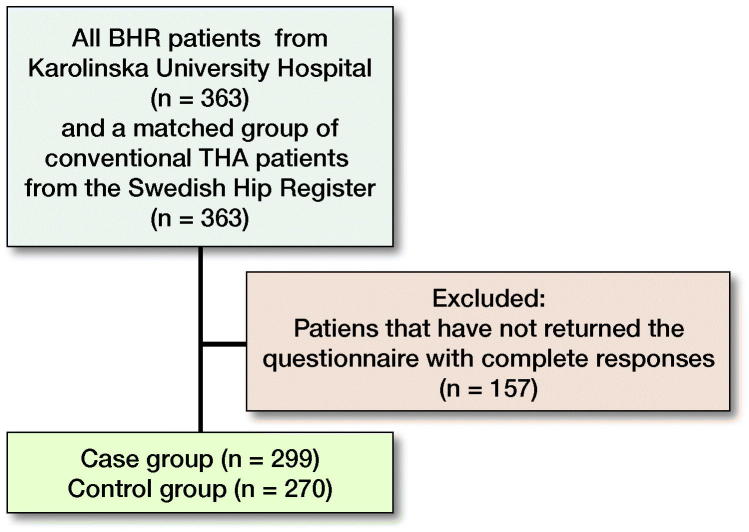
Graphic representation of postoperative PROMs after multivariable linear regression analyses. Bars represent 95% CI of the adjusted estimates (regression coefficients). For abbreviations, see Table 3.

Both the crude and adjusted estimates (Figure 2) showed that MoM-HR was associated with better scores in HOOS ADL (4.3, CI 1.8–6.9), and Sport/Rec (7.8, CI 3.8–12). We found no statistically significant association between type of prosthesis and remaining HOOS subscales, EQ-5D index, hip pain VAS, or satisfaction VAS.

## Discussion

Patients who underwent hip resurfacing reported better postoperative functional outcomes (HOOS subscales ADL and Sport/Rec) at mean 7 years post-surgery compared with a group of matched patients with conventional hip arthroplasty. We found no statistically significant differences in EQ-5D index, hip pain, or satisfaction. The largest difference between the groups was seen in the presumed most demanding subscale, i.e., function in sport and recreation.

Our observation is in accordance with the study of Haddad et al. (2015), showing that hip resurfacing yields better results regarding return to sports compared with conventional THA. The results also conform to a retrospective study of 215 resurfacing arthroplasties (mean F-U 2 years) (Girard et al. 2013), which showed that 41 of the 50 patients who participated in high-impact activity before the operation and onset of pain, returned to high-impact activity whilst 48 patients returned to any kind of physical activity. Although the last-mentioned study did not include a control group, other studies have demonstrated that only up to 40% of high-activity patients return to sport activity after conventional THA (Del Piccolo et al. 2016, Schmidutz et al. 2012).

When functional outcome scores were compared prospectively in 89 consecutively operated hips it was found that the resurfacing patients had greater improvement in Harris Hip scores, in UCLA activity score, and had a higher postoperative UCLA activity score than those operated with conventional THA (Fowble et al. 2009). On the other hand, the groups were not matched regarding overall health or preoperative functional outcome scores.

Tan et al. (2015) found that functional outcome scores and activity level from short to long-term follow-up were time-dependent. Among 100 patients with unilateral MoM-HR, they reported UCLA and SF-12 scores preoperatively, in the short term (mean F-U 2 years), and at a minimum of 10 years after the operation (mean F-U 12 years). They found no decrease in UCLA pain and walking scores between short-term and long-term follow-up, but a decrease in function and activity scores. With this in mind, when evaluating functional outcomes after hip arthroplasty, the results do not seem to be dependent only on functional outcome validation instruments, age, and sex but also on the time of the follow-up.

There are only a few previous studies comparing functional outcome scores between hip resurfacing and THA patients (Pollard et al. 2006, Mont et al. 2009, Costa et al. 2012). A retrospectively matched (sex, age, BMI, and activity level) study with a 7-year follow-up showed no difference in Oxford Hip Score but a higher level of activity as measured by UCLA score, and higher percentage (7% MoM-HR vs. 33% conventional THA) of patients participating in sports in the MoM-HR group (Pollard et al. 2006). Despite matching and medium–long follow-up, that study consisted of a rather small group of patients (53 MoM-HRs, 51 conventional THAs) making it difficult to draw certain conclusions. In another matched case-control study comprising 100 patients (50 MoM-HRs, 50 conventional THAs), the authors found no differences in mean Harris Hip Score (90 HR vs. 91 THA) or in patient satisfaction scores (9.2 HR vs. 8.8 THA) in short-term follow-up (Mont et al. 2009). As Harris Hip Score is limited to functional criteria, such a measure does not give an appropriate description of the patients’ functional outcome. In an assessor-blinded randomized controlled study (Costa et al. 2012) with 1:1 treatment allocation, hip function was similar between MoM-HR and THA at 12 months’ follow-up as measured with Harris Hip Score (88 MoM-HR vs. 82 THA) and Oxford Hip Score (40 MoM-HR vs. 38 THA). Furthermore, disability rating and activity level were similar in the first year after surgery. In that study, the long-term effects of HR were not studied. In the meantime, a 5-year F-U report is available that also shows similar hip function or health-related quality of life following a total hip arthroplasty vs. hip resurfacing (Costa et al. 2018).

When analyzing the “Forgotten Joint” Score-12 (78 MoM-HR vs. 76 THA) between MoM-HR and conventional THA, it was concluded that the choice of implant should not be based solely on any expectation that either yields superior clinical outcomes compared with the other at short-term follow-up (Ortiz-Declet et al. 2017).

Our study has some limitations. The collecting of PROMs did not reach nationwide coverage until 2008, which explains why preoperative data were not available for all of the patients (n = 157 had missing data preoperatively). However, missing preoperative EQ-5D data were successfully imputed and the EQ-5D scores were subsequently used for case-mix adjustment based on preoperative health status. Another limitation pertains to the lack of prospective HOOS data. Although groups were matched based on demography and baseline EQ-5D index, level of functioning in ADL and sports and recreation may have differed preoperatively. The occurrence of reoperations could be a potential source of bias albeit repeat surgeries were evenly distributed between the groups.

Whilst conventional THA is performed in most orthopedic units in Sweden, hip resurfacing was only performed in a few specialist centers during the study period. Therefore, all patients operated with HR either actively searched for institutions performing resurfacing prosthesis or were referred from other orthopedic units. Patients operated with conventional THAs likely did not actively request a certain implant, suggesting a biased selection that cannot be adjusted for. Moreover, almost all HR surgeries were performed by 2 experienced surgeons following well-established principles of surgical innovation in contrast to the control group, which was selected from the registry not considering surgeon experience. It must be constantly emphasized that introduction of new devices should follow a systematic approach even if the theoretical basis or preclinical results are excellent. Recently, Reito et al. (2017) described the anti-stepwise introduction of metal-on-metal hip replacements.

The strengths of our study include the careful 1:1 matching of the groups for the various demographic factors, surgical approach, time of surgery, and preoperative EQ-5D scores, which reduced many confounding factors. Our study also comprised a fairly large number of patients in the groups and with a satisfactory response rate. To our knowledge no study comparing functional outcome scores between MoM-HR and conventional THA has been undertaken with such a large number of patients followed for a comparable period of time.

Although the type of hip prosthesis did not influence the level of satisfaction, postoperative pain relief, or quality of life, MoM-HR patients had better postoperative HOOS scores in the function of daily living and function in sports and recreation domains. Translating the adjusted regression estimates of these 2 HOOS subscales into effect sizes, the influence of MoM-HR was moderate (0.25 and 0.30, respectively). Furthermore, there was no statistically significant difference in reoperation rates using a Birmingham Hip Replacement (BHR) compared with a conventional implant in these 2 age- and sex-matched patient groups. As MoM-HR was developed to address the special demands of a younger and more active population, our results support the rationale for using the technique in this group of patients.

Choice of hip arthroplasty for young and active patients with high expectations is still challenging, mostly due to higher risks of wear, dislocation, and need of revision surgery. In summary, by comparing MoM-HR with conventional THA in a matched study design (mean 7 years F-U) of a selected group of patients we have shown MoM-HR to yield better functional outcome scores in 2/5 HOOS subscales; all other outcome measures were similar. When a BHR implant is considered, patients should be informed of the risk of developing of adverse reactions and uncertain long-term results. We highly recommend subsequent close follow-up for this matter.

### Supplementary data

Figure 1 and Table 2 are available as supplementary data in the online version of this article, http://dx.doi.org/10.1080/ 17453674.2019.1604343

OR, HH, and LFT conceived and designed the study. OR and HH obtained ethical approval. OR and SN collected data. SN performed statistical analysis. AO drafted the manuscript. All authors interpreted the results and reviewed, edited, and approved the final version of the manuscript.

*Acta* thanks Nina Mathijssen and Marc Nijhof for help with peer review of this study.

## Supplementary Material

Supplemental Material
