# Supplementary material for: Patient-reported outcomes in hip resurfacing versus conventional total hip arthroplasty: a register-based matched cohort study of 726 patients
Source: Acta Orthop. 2019 Apr 18;90(4):318–23. doi: 10.1080/17453674.2019.1604343 (PMC6718187; doi:10.1080/17453674.2019.1604343)
Supplement: Supplemental Material [file IORT_A_1604343_SM8149.pdf]

Supplementary data

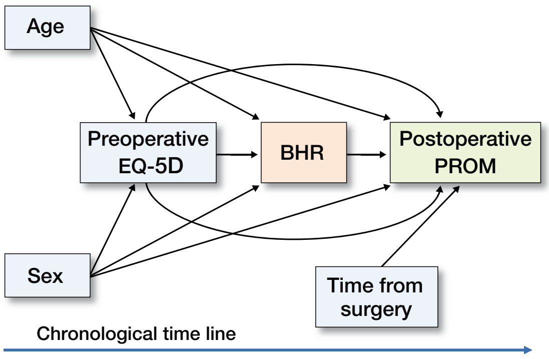

Figure A1. Directed Acyclic Graph (DAG) showing variables pathways and relation to exposure and outcome.

Table 2. Non-respondent analysis

Characteristics	Respondents	Non-respondents	p-value
Number of patients	569	157	
Women, n (%)	132 (23)	44 (28)	0.3
Cases, n (%)	299 (53)	64 (41)	0.01
Age at primary operation, mean (SD)	52 (8.6)	49 (9.0)	0.001
Year of surgery, mean (SD)	2008 (2.9)	2008 (2.8)	0.8
Distribution of diagnoses, n (%)			0.1
Primary osteoarthritis	508 (89)	132 (84)	
Childhood hip disease	49 (8.6)	23 (15)	
Other hip joint disorders	12 (2.1)	2 (1.3)	
PROMs preoperative			
VAS hip pain, mean (SD)	70 (18)	72 (19)	0.3
EQ-5D index, mean (SD)	0.49 (0.31)	0.40 (0.32)	0.006

SD = standard deviation; VAS = visual analog scale; EQ-5D = EuroQol 5 dimensions.
